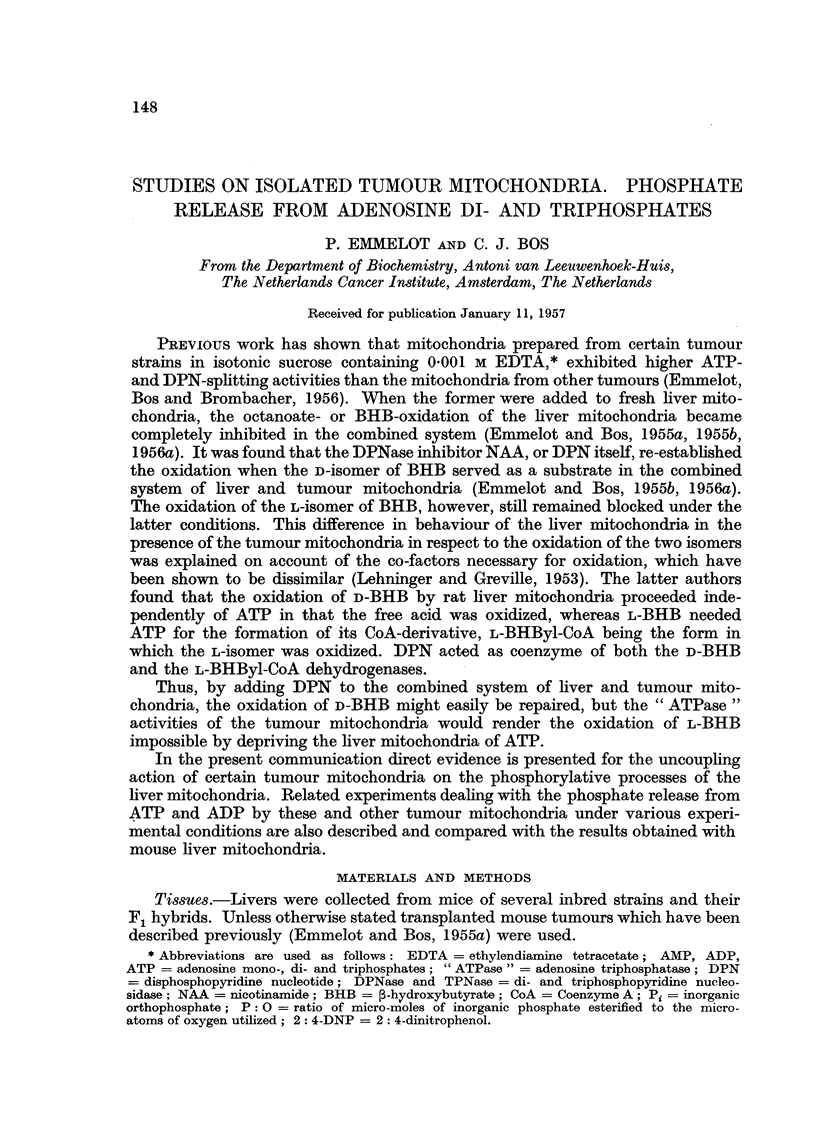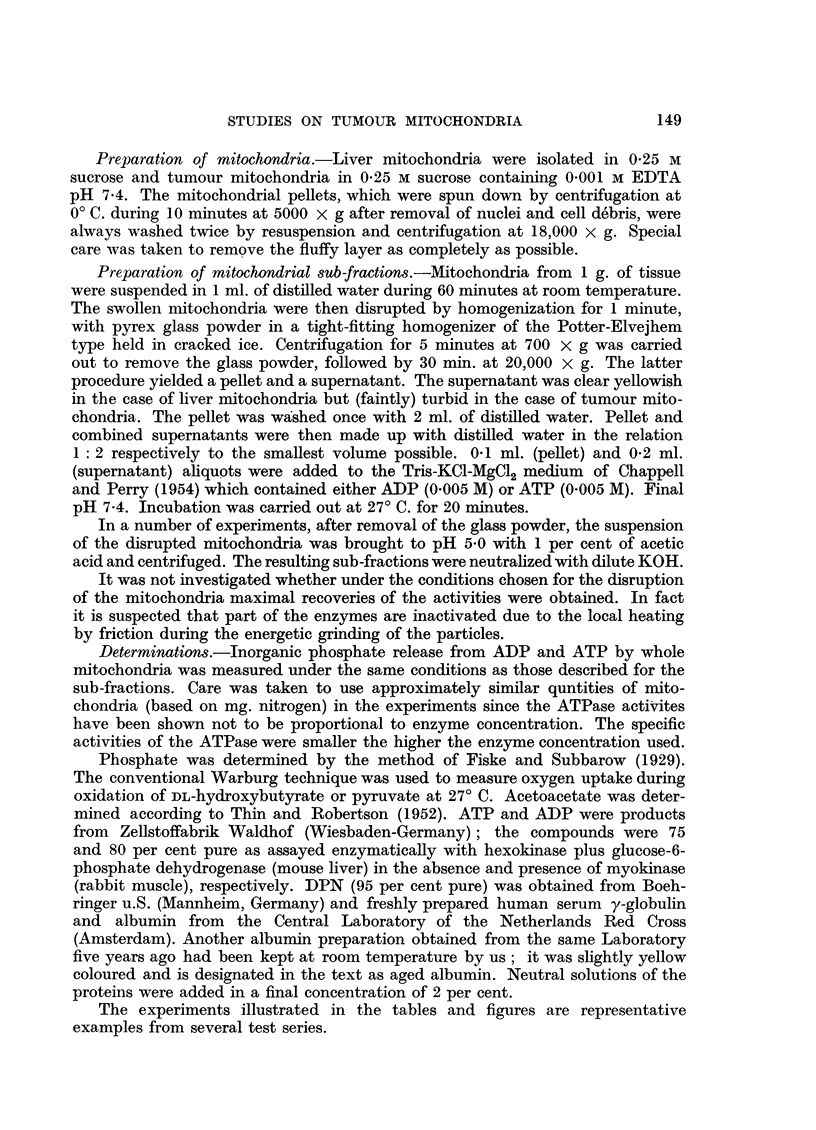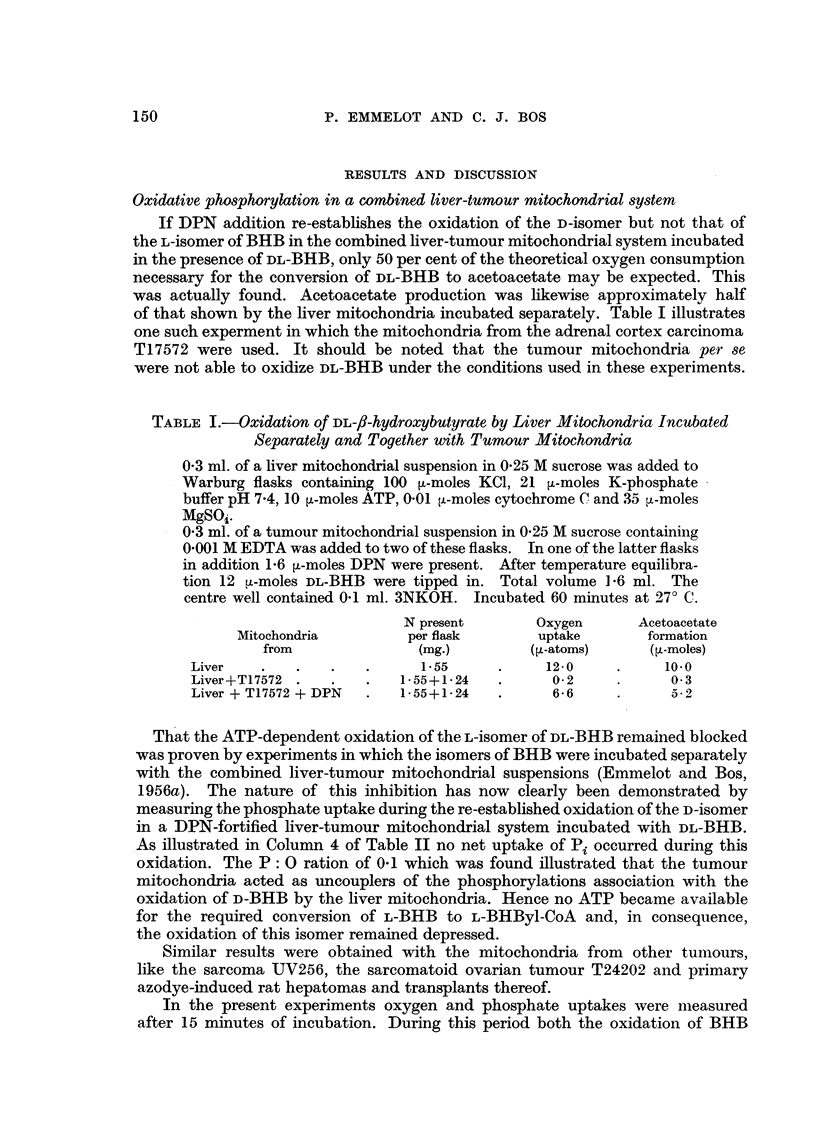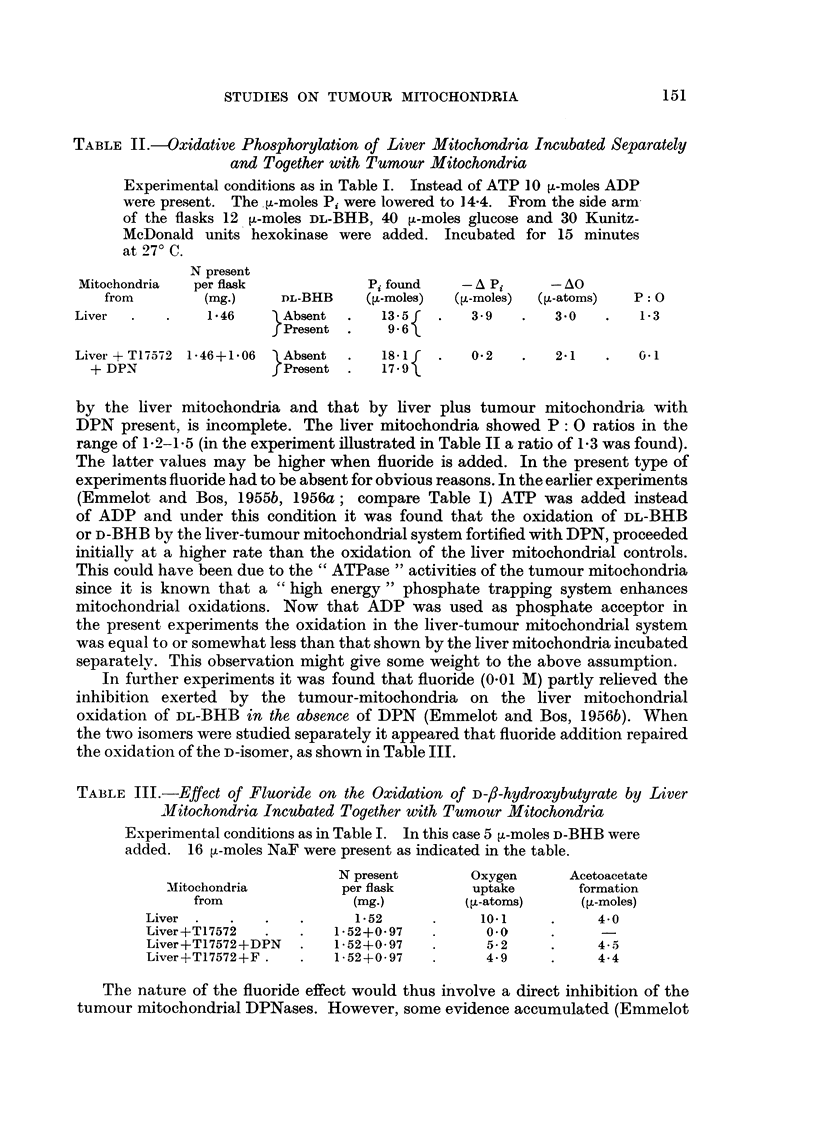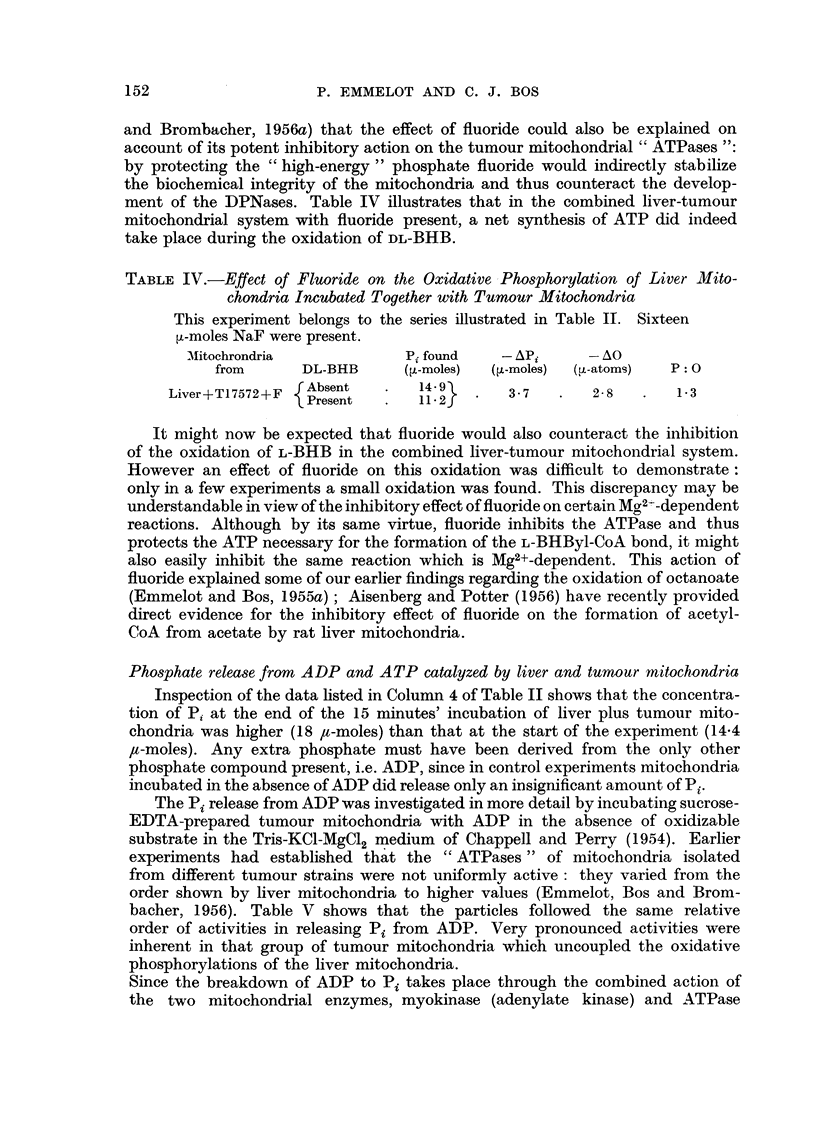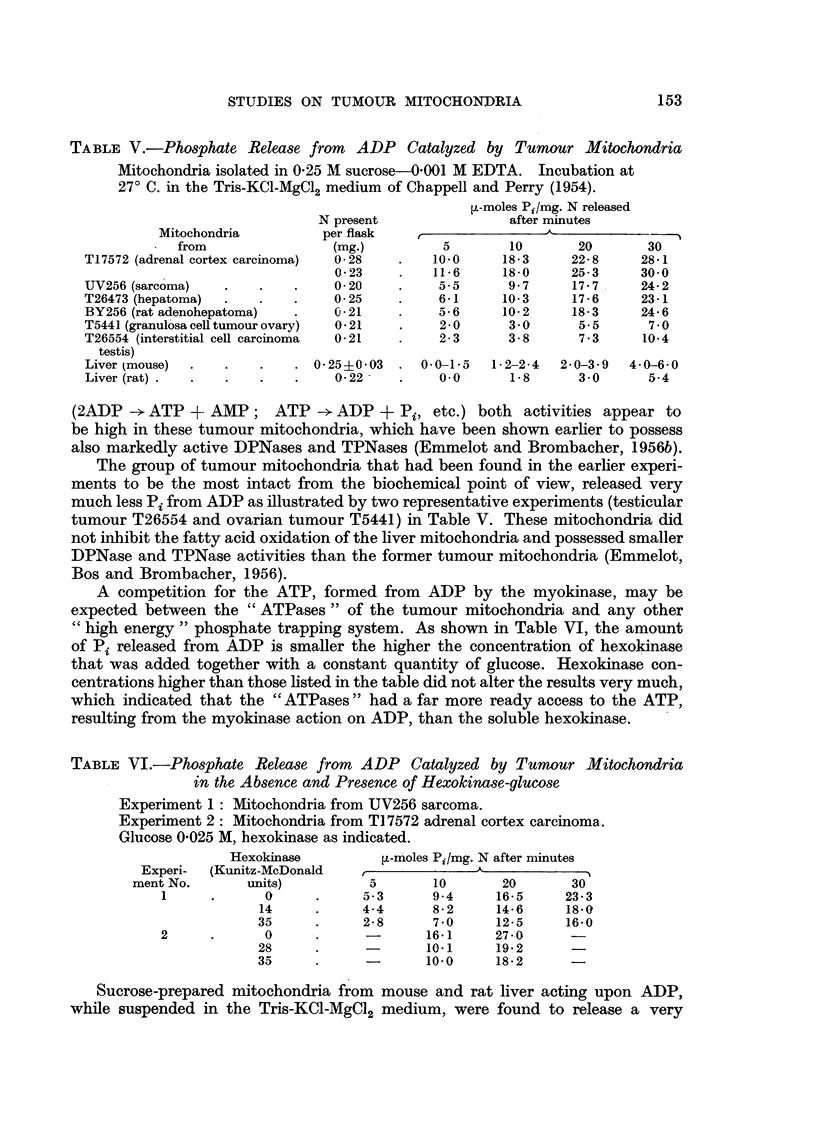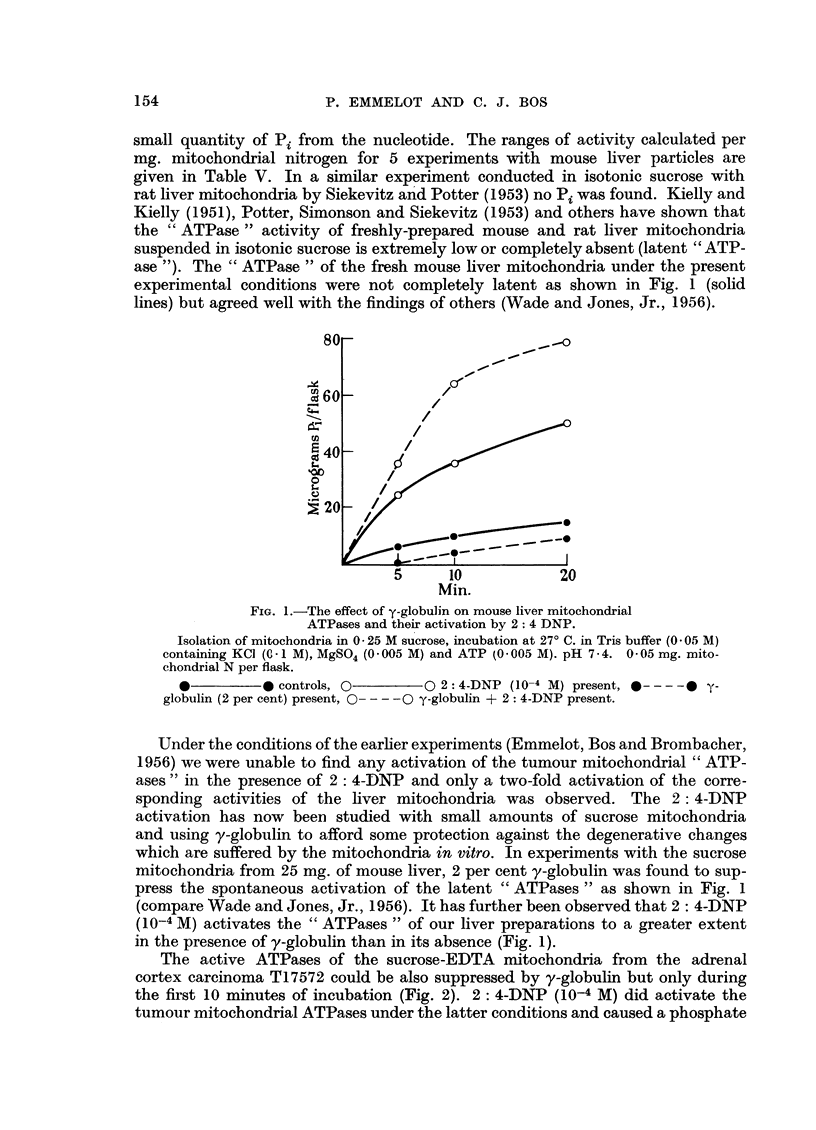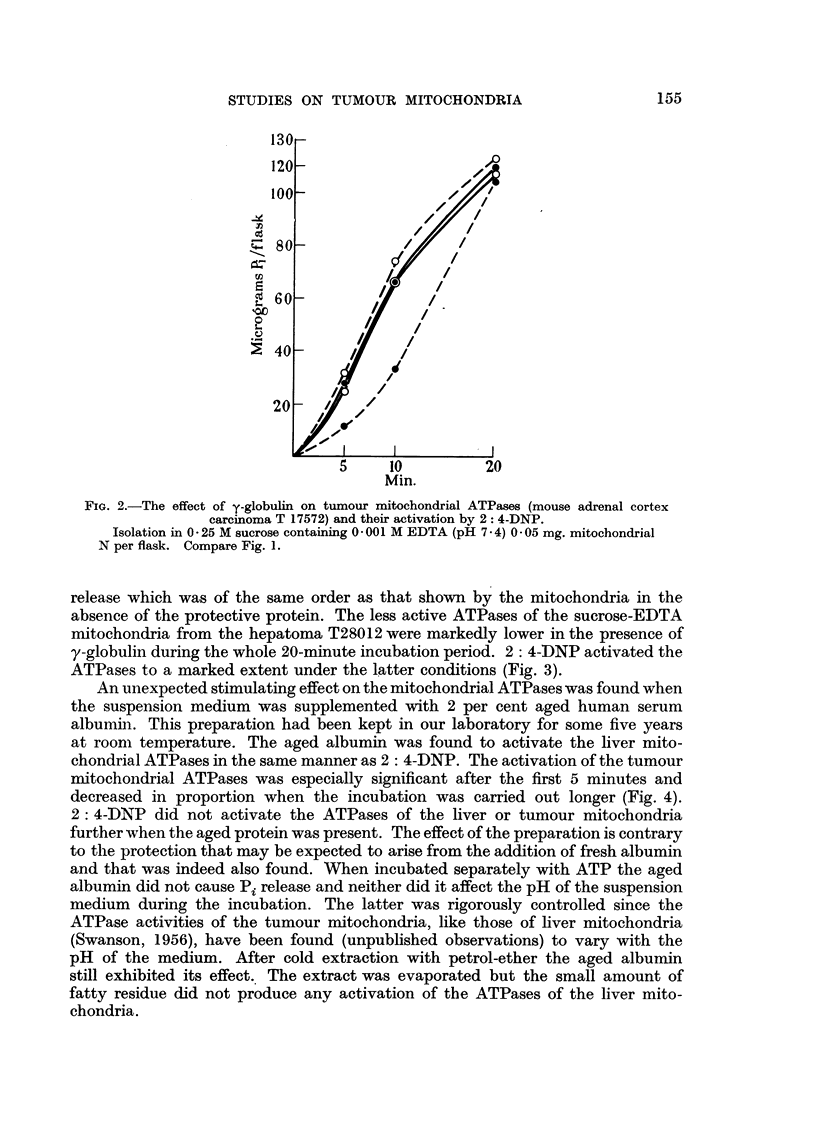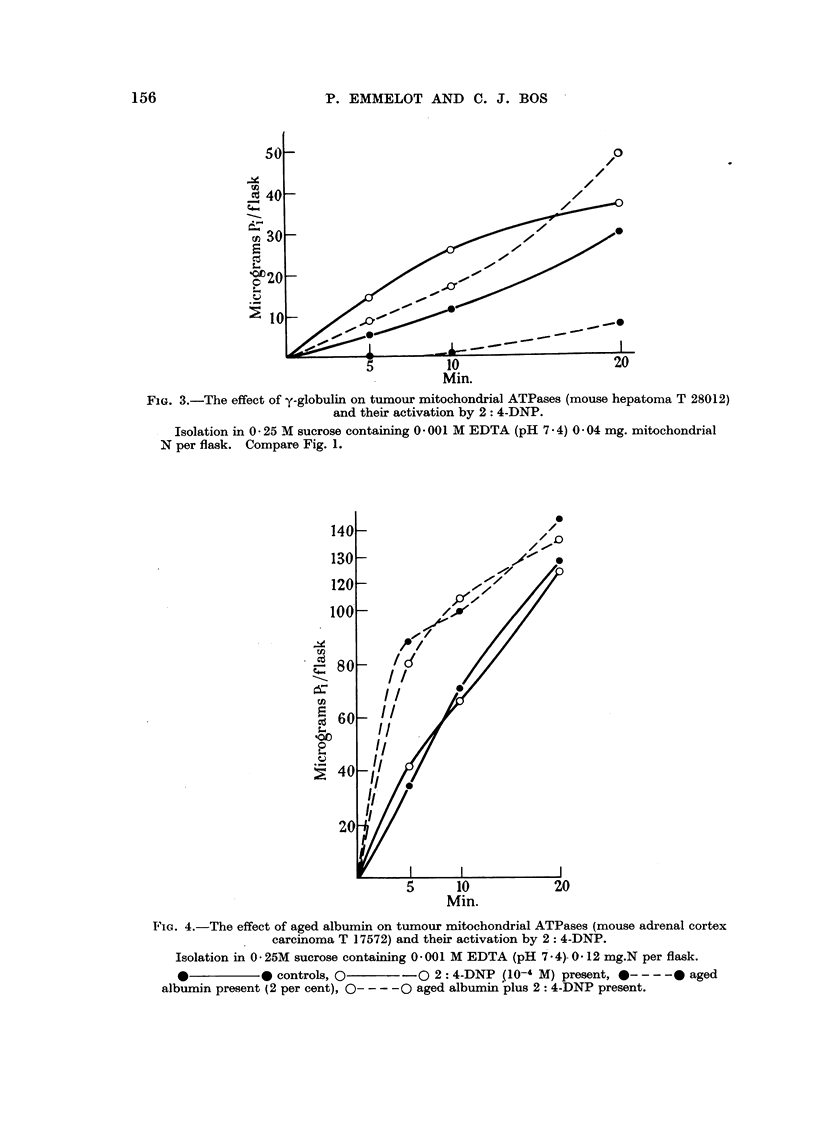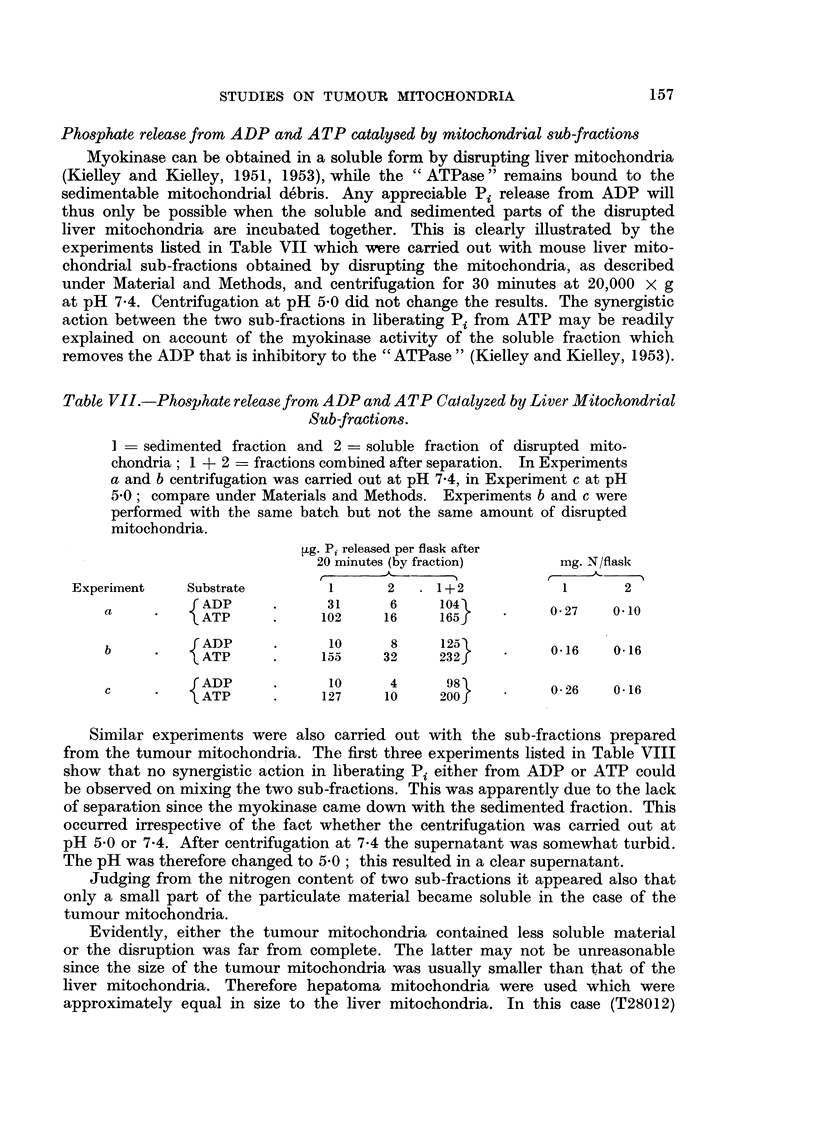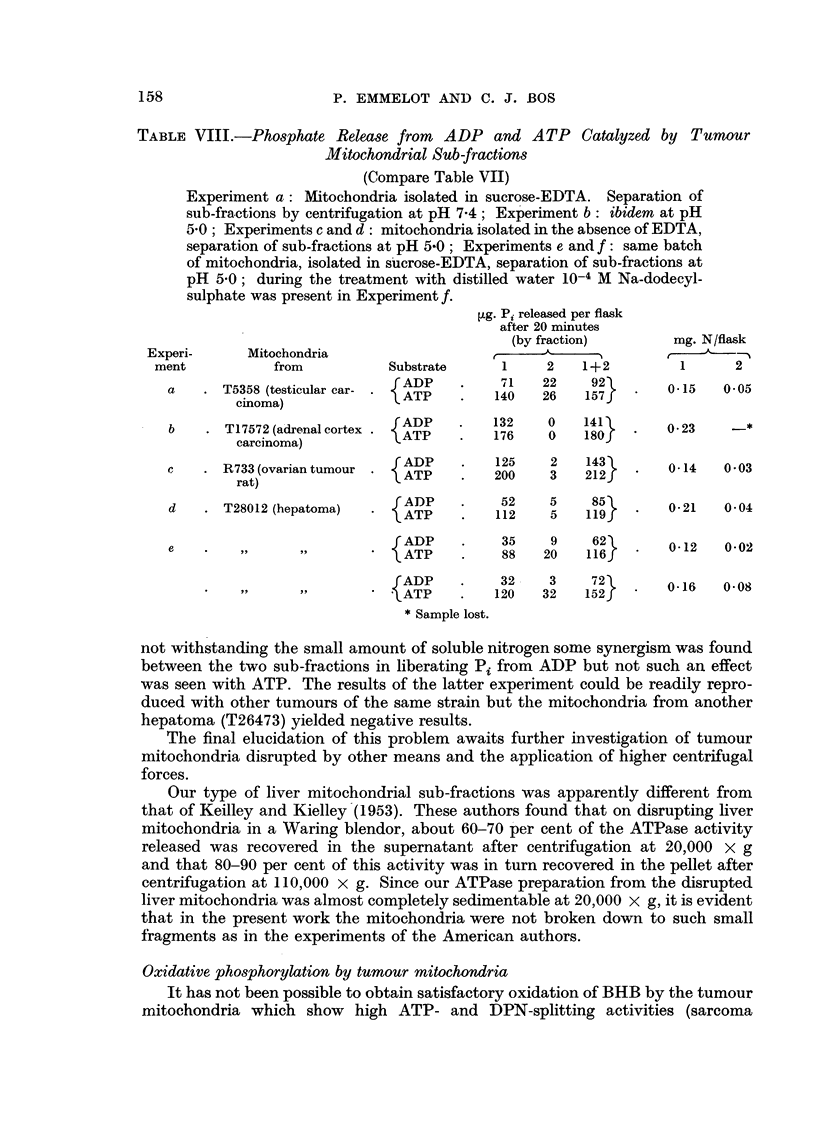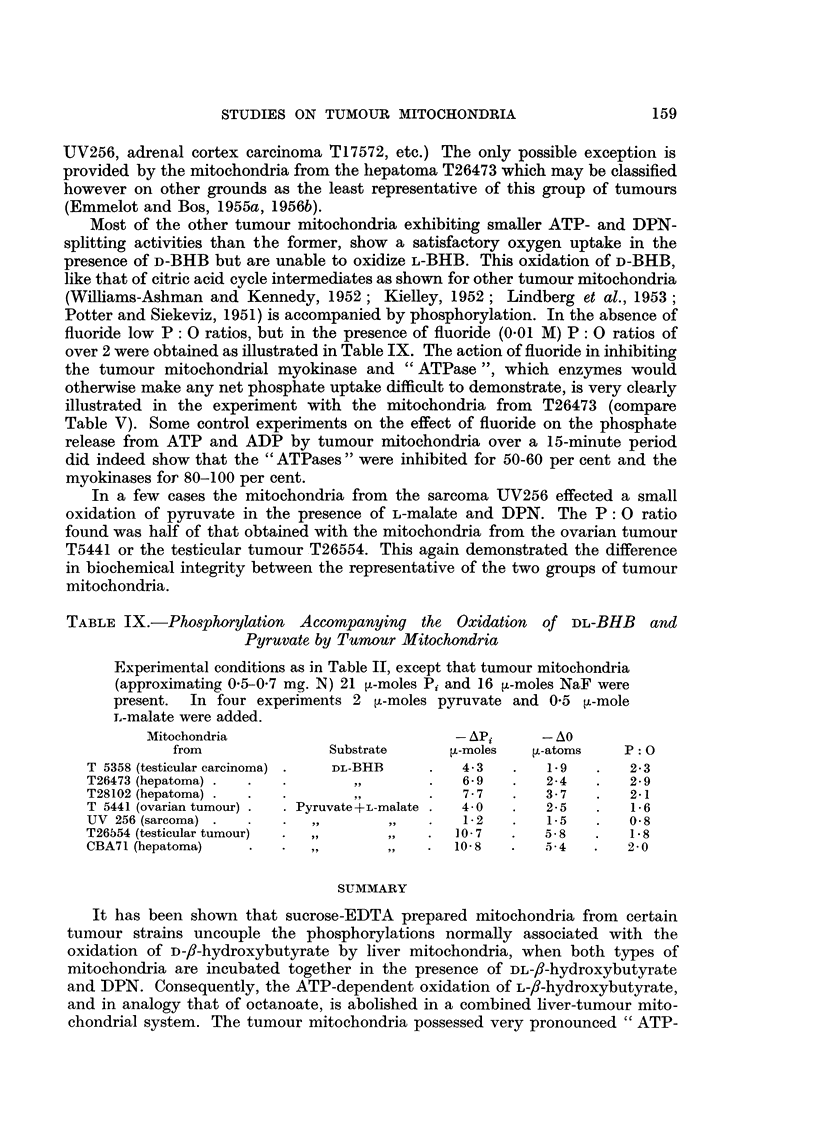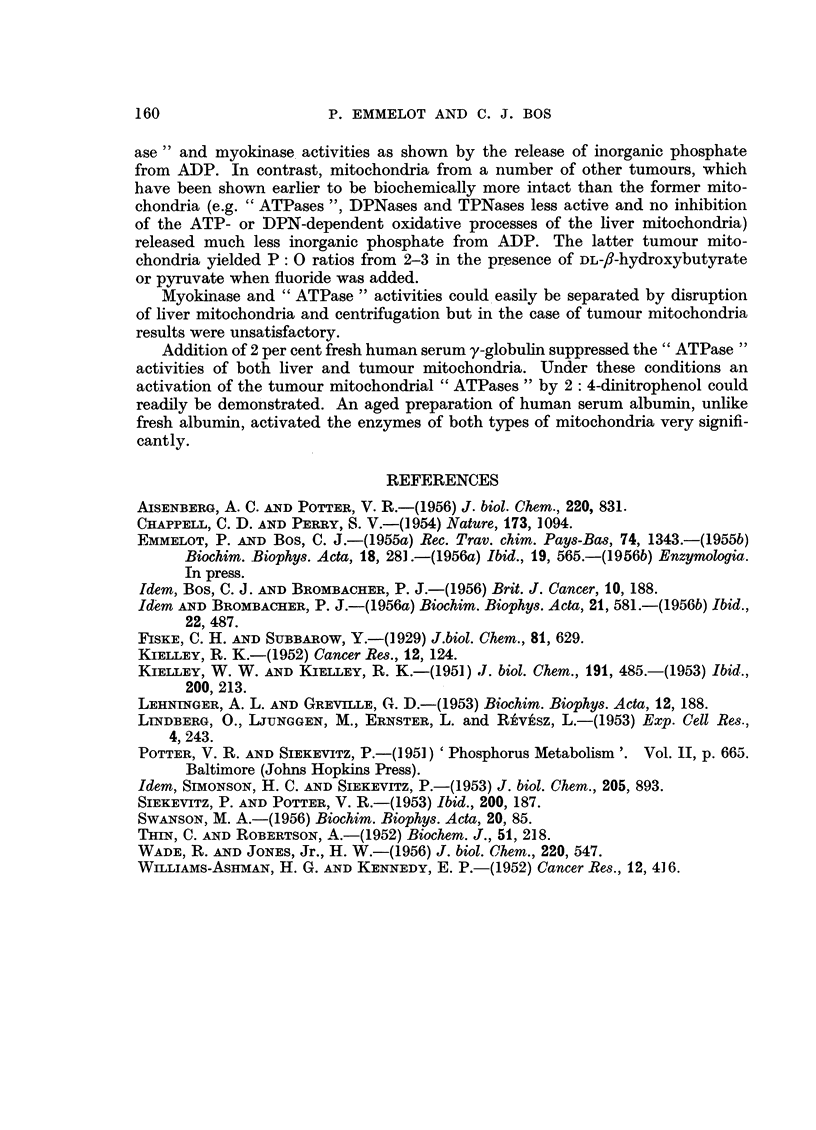# Studies on Isolated Tumour Mitochondria. Phosphate Release from Adenosine Di- and Triphosphates

**DOI:** 10.1038/bjc.1957.21

**Published:** 1957-03

**Authors:** P. Emmelot, C. J. Bos


### STUDIES ON ISOLATED TUMOUR MITOCHONDRIA. PHOSPHATE RELEASE FROM ADENOSINE DI- AND TRIPHOSPHATES

P. EMMELOT AND C. J. BOS

*From the Department of Biochemistry, Antoni van Leeuwenhoek-Huis, The Netherlands Cancer Institute, Amsterdam, The Netherlands*

Received for publication January 11, 1957

PREVIOUS work has shown that mitochondria prepared from certain tumour strains in isotonic sucrose containing 0·001 M EDTA,* exhibited higher ATP- and DPN-splitting activities than the mitochondria from other tumours (Emmelot, Bos and Brombacher, 1956). When the former were added to fresh liver mitochondria, the octanoate- or BHB-oxidation of the liver mitochondria became completely inhibited in the combined system (Emmelot and Bos, 1955*a*, 1955*b*, 1956*a*). It was found that the DPNase inhibitor NAA, or DPN itself, re-established the oxidation when the D-isomer of BHB served as a substrate in the combined system of liver and tumour mitochondria (Emmelot and Bos, 1955*b*, 1956*a*). The oxidation of the L-isomer of BHB, however, still remained blocked under the latter conditions. This difference in behaviour of the liver mitochondria in the presence of the tumour mitochondria in respect to the oxidation of the two isomers was explained on account of the co-factors necessary for oxidation, which have been shown to be dissimilar (Lehninger and Greville, 1953). The latter authors found that the oxidation of D-BHB by rat liver mitochondria proceeded independently of ATP in that the free acid was oxidized, whereas L-BHB needed ATP for the formation of its CoA-derivative, L-BHByl-CoA being the form in which the L-isomer was oxidized. DPN acted as coenzyme of both the D-BHB and the L-BHByl-CoA dehydrogenases.

Thus, by adding DPN to the combined system of liver and tumour mitochondria, the oxidation of D-BHB might easily be repaired, but the " ATPase " activities of the tumour mitochondria would render the oxidation of L-BHB impossible by depriving the liver mitochondria of ATP.

In the present communication direct evidence is presented for the uncoupling action of certain tumour mitochondria on the phosphorylative processes of the liver mitochondria. Related experiments dealing with the phosphate release from ATP and ADP by these and other tumour mitochondria under various experimental conditions are also described and compared with the results obtained with mouse liver mitochondria.

### MATERIALS AND METHODS

*Tissues.*—Livers were collected from mice of several inbred strains and their $F_1$ hybrids. Unless otherwise stated transplanted mouse tumours which have been described previously (Emmelot and Bos, 1955*a*) were used.

* Abbreviations are used as follows: EDTA = ethylendiamine tetracetate; AMP, ADP, ATP = adenosine mono-, di- and triphosphates; " ATPase " = adenosine triphosphatase; DPN = disphosphopyridine nucleotide; DPNase and TPNase = di- and triphosphopyridine nucleosidase; NAA = nicotinamide; BHB = β-hydroxybutyrate; CoA = Coenzyme A; $P_i$ = inorganic orthophosphate; P : O = ratio of micro-moles of inorganic phosphate esterified to the micro-atoms of oxygen utilized; 2 : 4-DNP = 2 : 4-dinitrophenol.

*Preparation of mitochondria.*—Liver mitochondria were isolated in 0·25 M sucrose and tumour mitochondria in 0·25 M sucrose containing 0·001 M EDTA pH 7·4. The mitochondrial pellets, which were spun down by centrifugation at 0° C. during 10 minutes at 5000 × g after removal of nuclei and cell débris, were always washed twice by resuspension and centrifugation at 18,000 × g. Special care was taken to remove the fluffy layer as completely as possible.

*Preparation of mitochondrial sub-fractions.*—Mitochondria from 1 g. of tissue were suspended in 1 ml. of distilled water during 60 minutes at room temperature. The swollen mitochondria were then disrupted by homogenization for 1 minute, with pyrex glass powder in a tight-fitting homogenizer of the Potter-Elvejhem type held in cracked ice. Centrifugation for 5 minutes at 700 × g was carried out to remove the glass powder, followed by 30 min. at 20,000 × g. The latter procedure yielded a pellet and a supernatant. The supernatant was clear yellowish in the case of liver mitochondria but (faintly) turbid in the case of tumour mitochondria. The pellet was washed once with 2 ml. of distilled water. Pellet and combined supernatants were then made up with distilled water in the relation 1 : 2 respectively to the smallest volume possible. 0·1 ml. (pellet) and 0·2 ml. (supernatant) aliquots were added to the Tris-KCl-$MgCl_2$ medium of Chappell and Perry (1954) which contained either ADP (0·005 M) or ATP (0·005 M). Final pH 7·4. Incubation was carried out at 27° C. for 20 minutes.

In a number of experiments, after removal of the glass powder, the suspension of the disrupted mitochondria was brought to pH 5·0 with 1 per cent of acetic acid and centrifuged. The resulting sub-fractions were neutralized with dilute KOH.

It was not investigated whether under the conditions chosen for the disruption of the mitochondria maximal recoveries of the activities were obtained. In fact it is suspected that part of the enzymes are inactivated due to the local heating by friction during the energetic grinding of the particles.

*Determinations.*—Inorganic phosphate release from ADP and ATP by whole mitochondria was measured under the same conditions as those described for the sub-fractions. Care was taken to use approximately similar quntities of mitochondria (based on mg. nitrogen) in the experiments since the ATPase activites have been shown not to be proportional to enzyme concentration. The specific activities of the ATPase were smaller the higher the enzyme concentration used.

Phosphate was determined by the method of Fiske and Subbarow (1929). The conventional Warburg technique was used to measure oxygen uptake during oxidation of DL-hydroxybutyrate or pyruvate at 27° C. Acetoacetate was determined according to Thin and Robertson (1952). ATP and ADP were products from Zellstoffabrik Waldhof (Wiesbaden-Germany); the compounds were 75 and 80 per cent pure as assayed enzymatically with hexokinase plus glucose-6-phosphate dehydrogenase (mouse liver) in the absence and presence of myokinase (rabbit muscle), respectively. DPN (95 per cent pure) was obtained from Boehringer u.S. (Mannheim, Germany) and freshly prepared human serum $\gamma$-globulin and albumin from the Central Laboratory of the Netherlands Red Cross (Amsterdam). Another albumin preparation obtained from the same Laboratory five years ago had been kept at room temperature by us; it was slightly yellow coloured and is designated in the text as aged albumin. Neutral solutions of the proteins were added in a final concentration of 2 per cent.

The experiments illustrated in the tables and figures are representative examples from several test series.

### RESULTS AND DISCUSSION

### *Oxidative phosphorylation in a combined liver-tumour mitochondrial system*

If DPN addition re-establishes the oxidation of the D-isomer but not that of the L-isomer of BHB in the combined liver-tumour mitochondrial system incubated in the presence of DL-BHB, only 50 per cent of the theoretical oxygen consumption necessary for the conversion of DL-BHB to acetoacetate may be expected. This was actually found. Acetoacetate production was likewise approximately half of that shown by the liver mitochondria incubated separately. Table I illustrates one such experment in which the mitochondria from the adrenal cortex carcinoma T17572 were used. It should be noted that the tumour mitochondria *per se* were not able to oxidize DL-BHB under the conditions used in these experiments.

TABLE I.—*Oxidation of* DL-*β-hydroxybutyrate by Liver Mitochondria Incubated Separately and Together with Tumour Mitochondria*

0·3 ml. of a liver mitochondrial suspension in 0·25 M sucrose was added to Warburg flasks containing 100 μ-moles KCl, 21 μ-moles K-phosphate buffer pH 7·4, 10 μ-moles ATP, 0·01 μ-moles cytochrome C and 35 μ-moles $MgSO_4$.
0·3 ml. of a tumour mitochondrial suspension in 0·25 M sucrose containing 0·001 M EDTA was added to two of these flasks. In one of the latter flasks in addition 1·6 μ-moles DPN were present. After temperature equilibration 12 μ-moles DL-BHB were tipped in. Total volume 1·6 ml. The centre well contained 0·1 ml. 3NKOH. Incubated 60 minutes at 27° C.

| Mitochondria from | N present per flask (mg.) | Oxygen uptake (μ-atoms) | Acetoacetate formation (μ-moles) |
|---|---|---|---|
| Liver | 1·55 | 12·0 | 10·0 |
| Liver+T17572 | 1·55+1·24 | 0·2 | 0·3 |
| Liver + T17572 + DPN | 1·55+1·24 | 6·6 | 5·2 |

That the ATP-dependent oxidation of the L-isomer of DL-BHB remained blocked was proven by experiments in which the isomers of BHB were incubated separately with the combined liver-tumour mitochondrial suspensions (Emmelot and Bos, 1956*a*). The nature of this inhibition has now clearly been demonstrated by measuring the phosphate uptake during the re-established oxidation of the D-isomer in a DPN-fortified liver-tumour mitochondrial system incubated with DL-BHB. As illustrated in Column 4 of Table II no net uptake of $P_i$ occurred during this oxidation. The P : O ration of 0·1 which was found illustrated that the tumour mitochondria acted as uncouplers of the phosphorylations association with the oxidation of D-BHB by the liver mitochondria. Hence no ATP became available for the required conversion of L-BHB to L-BHByl-CoA and, in consequence, the oxidation of this isomer remained depressed.

Similar results were obtained with the mitochondria from other tumours, like the sarcoma UV256, the sarcomatoid ovarian tumour T24202 and primary azodye-induced rat hepatomas and transplants thereof.

In the present experiments oxygen and phosphate uptakes were measured after 15 minutes of incubation. During this period both the oxidation of BHB

TABLE II.—*Oxidative Phosphorylation of Liver Mitochondria Incubated Separately and Together with Tumour Mitochondria*

Experimental conditions as in Table I. Instead of ATP 10 μ-moles ADP were present. The μ-moles $P_i$ were lowered to 14·4. From the side arm of the flasks 12 μ-moles DL-BHB, 40 μ-moles glucose and 30 Kunitz-McDonald units hexokinase were added. Incubated for 15 minutes at 27° C.

| Mitochondria from | N present per flask (mg.) | DL-BHB | $P_i$ found (μ-moles) | $-\Delta P_i$ (μ-moles) | $-\Delta O$ (μ-atoms) | P : O |
|---|---|---|---|---|---|---|
| Liver | 1·46 | Absent | 13·5 | 3·9 | 3·0 | 1·3 |
| | | Present | 9·6 | | | |
| Liver + T17572 + DPN | 1·46+1·06 | Absent | 18·1 | 0·2 | 2·1 | 0·1 |
| | | Present | 17·9 | | | |

by the liver mitochondria and that by liver plus tumour mitochondria with DPN present, is incomplete. The liver mitochondria showed P : O ratios in the range of 1·2–1·5 (in the experiment illustrated in Table II a ratio of 1·3 was found). The latter values may be higher when fluoride is added. In the present type of experiments fluoride had to be absent for obvious reasons. In the earlier experiments (Emmelot and Bos, 1955*b*, 1956*a*; compare Table I) ATP was added instead of ADP and under this condition it was found that the oxidation of DL-BHB or D-BHB by the liver-tumour mitochondrial system fortified with DPN, proceeded initially at a higher rate than the oxidation of the liver mitochondrial controls. This could have been due to the " ATPase " activities of the tumour mitochondria since it is known that a " high energy " phosphate trapping system enhances mitochondrial oxidations. Now that ADP was used as phosphate acceptor in the present experiments the oxidation in the liver-tumour mitochondrial system was equal to or somewhat less than that shown by the liver mitochondria incubated separately. This observation might give some weight to the above assumption.

In further experiments it was found that fluoride (0·01 M) partly relieved the inhibition exerted by the tumour-mitochondria on the liver mitochondrial oxidation of DL-BHB *in the absence* of DPN (Emmelot and Bos, 1956*b*). When the two isomers were studied separately it appeared that fluoride addition repaired the oxidation of the D-isomer, as shown in Table III.

TABLE III.—*Effect of Fluoride on the Oxidation of* D-*β-hydroxybutyrate by Liver Mitochondria Incubated Together with Tumour Mitochondria*

Experimental conditions as in Table I. In this case 5 μ-moles D-BHB were added. 16 μ-moles NaF were present as indicated in the table.

| Mitochondria from | N present per flask (mg.) | Oxygen uptake (μ-atoms) | Acetoacetate formation (μ-moles) |
|---|---|---|---|
| Liver | 1·52 | 10·1 | 4·0 |
| Liver+T17572 | 1·52+0·97 | 0·0 | — |
| Liver+T17572+DPN | 1·52+0·97 | 5·2 | 4·5 |
| Liver+T17572+F | 1·52+0·97 | 4·9 | 4·4 |

The nature of the fluoride effect would thus involve a direct inhibition of the tumour mitochondrial DPNases. However, some evidence accumulated (Emmelot and Brombacher, 1956*a*) that the effect of fluoride could also be explained on account of its potent inhibitory action on the tumour mitochondrial " ATPases ": by protecting the " high-energy " phosphate fluoride would indirectly stabilize the biochemical integrity of the mitochondria and thus counteract the development of the DPNases. Table IV illustrates that in the combined liver-tumour mitochondrial system with fluoride present, a net synthesis of ATP did indeed take place during the oxidation of DL-BHB.

TABLE IV.—*Effect of Fluoride on the Oxidative Phosphorylation of Liver Mitochondria Incubated Together with Tumour Mitochondria*

This experiment belongs to the series illustrated in Table II. Sixteen $\mu$-moles NaF were present.

| Mitochrondria from | DL-BHB | $P_i$ found ($\mu$-moles) | $-\Delta P_i$ ($\mu$-moles) | $-\Delta O$ ($\mu$-atoms) | P : O |
|---|---|---|---|---|---|
| Liver+T17572+F | Absent | 14·9 | 3·7 | 2·8 | 1·3 |
| | Present | 11·2 | | | |

It might now be expected that fluoride would also counteract the inhibition of the oxidation of L-BHB in the combined liver-tumour mitochondrial system. However an effect of fluoride on this oxidation was difficult to demonstrate: only in a few experiments a small oxidation was found. This discrepancy may be understandable in view of the inhibitory effect of fluoride on certain $Mg^{2+}$-dependent reactions. Although by its same virtue, fluoride inhibits the ATPase and thus protects the ATP necessary for the formation of the L-BHByl-CoA bond, it might also easily inhibit the same reaction which is $Mg^{2+}$-dependent. This action of fluoride explained some of our earlier findings regarding the oxidation of octanoate (Emmelot and Bos, 1955*a*); Aisenberg and Potter (1956) have recently provided direct evidence for the inhibitory effect of fluoride on the formation of acetyl-CoA from acetate by rat liver mitochondria.

*Phosphate release from ADP and ATP catalyzed by liver and tumour mitochondria*

Inspection of the data listed in Column 4 of Table II shows that the concentration of $P_i$ at the end of the 15 minutes' incubation of liver plus tumour mitochondria was higher (18 $\mu$-moles) than that at the start of the experiment (14·4 $\mu$-moles). Any extra phosphate must have been derived from the only other phosphate compound present, i.e. ADP, since in control experiments mitochondria incubated in the absence of ADP did release only an insignificant amount of $P_i$.

The $P_i$ release from ADP was investigated in more detail by incubating sucrose-EDTA-prepared tumour mitochondria with ADP in the absence of oxidizable substrate in the Tris-KCl-$MgCl_2$ medium of Chappell and Perry (1954). Earlier experiments had established that the " ATPases " of mitochondria isolated from different tumour strains were not uniformly active: they varied from the order shown by liver mitochondria to higher values (Emmelot, Bos and Brombacher, 1956). Table V shows that the particles followed the same relative order of activities in releasing $P_i$ from ADP. Very pronounced activities were inherent in that group of tumour mitochondria which uncoupled the oxidative phosphorylations of the liver mitochondria.

Since the breakdown of ADP to $P_i$ takes place through the combined action of the two mitochondrial enzymes, myokinase (adenylate kinase) and ATPase

TABLE V.—*Phosphate Release from ADP Catalyzed by Tumour Mitochondria*

Mitochondria isolated in 0·25 M sucrose—0·001 M EDTA. Incubation at 27° C. in the Tris-KCl-$MgCl_2$ medium of Chappell and Perry (1954).

| Mitochondria from | N present per flask (mg.) | $\mu$-moles $P_i$/mg. N released after minutes 5 | 10 | 20 | 30 |
|---|---|---|---|---|---|
| T17572 (adrenal cortex carcinoma) | 0·28 | 10·0 | 18·3 | 22·8 | 28·1 |
| | 0·23 | 11·6 | 18·0 | 25·3 | 30·0 |
| UV256 (sarcoma) | 0·20 | 5·5 | 9·7 | 17·7 | 24·2 |
| T26473 (hepatoma) | 0·25 | 6·1 | 10·3 | 17·6 | 23·1 |
| BY256 (rat adenohepatoma) | 0·21 | 5·6 | 10·2 | 18·3 | 24·6 |
| T5441 (granulosa cell tumour ovary) | 0·21 | 2·0 | 3·0 | 5·5 | 7·0 |
| T26554 (interstitial cell carcinoma testis) | 0·21 | 2·3 | 3·8 | 7·3 | 10·4 |
| Liver (mouse) | 0·25±0·03 | 0·0–1·5 | 1·2–2·4 | 2·0–3·9 | 4·0–6·0 |
| Liver (rat) | 0·22 | 0·0 | 1·8 | 3·0 | 5·4 |

(2ADP → ATP + AMP ; ATP → ADP + $P_i$, etc.) both activities appear to be high in these tumour mitochondria, which have been shown earlier to possess also markedly active DPNases and TPNases (Emmelot and Brombacher, 1956*b*).

The group of tumour mitochondria that had been found in the earlier experiments to be the most intact from the biochemical point of view, released very much less $P_i$ from ADP as illustrated by two representative experiments (testicular tumour T26554 and ovarian tumour T5441) in Table V. These mitochondria did not inhibit the fatty acid oxidation of the liver mitochondria and possessed smaller DPNase and TPNase activities than the former tumour mitochondria (Emmelot, Bos and Brombacher, 1956).

A competition for the ATP, formed from ADP by the myokinase, may be expected between the " ATPases " of the tumour mitochondria and any other " high energy " phosphate trapping system. As shown in Table VI, the amount of $P_i$ released from ADP is smaller the higher the concentration of hexokinase that was added together with a constant quantity of glucose. Hexokinase concentrations higher than those listed in the table did not alter the results very much, which indicated that the "ATPases" had a far more ready access to the ATP, resulting from the myokinase action on ADP, than the soluble hexokinase.

TABLE VI.—*Phosphate Release from ADP Catalyzed by Tumour Mitochondria in the Absence and Presence of Hexokinase-glucose*

Experiment 1 : Mitochondria from UV256 sarcoma.
Experiment 2 : Mitochondria from T17572 adrenal cortex carcinoma.
Glucose 0·025 M, hexokinase as indicated.

| Experiment No. | Hexokinase (Kunitz-McDonald units) | $\mu$-moles $P_i$/mg. N after minutes 5 | 10 | 20 | 30 |
|---|---|---|---|---|---|
| 1 | 0 | 5·3 | 9·4 | 16·5 | 23·3 |
| | 14 | 4·4 | 8·2 | 14·6 | 18·0 |
| | 35 | 2·8 | 7·0 | 12·5 | 16·0 |
| 2 | 0 | — | 16·1 | 27·0 | — |
| | 28 | — | 10·1 | 19·2 | — |
| | 35 | — | 10·0 | 18·2 | — |

Sucrose-prepared mitochondria from mouse and rat liver acting upon ADP, while suspended in the Tris-KCl-$MgCl_2$ medium, were found to release a very small quantity of $P_i$ from the nucleotide. The ranges of activity calculated per mg. mitochondrial nitrogen for 5 experiments with mouse liver particles are given in Table V. In a similar experiment conducted in isotonic sucrose with rat liver mitochondria by Siekevitz and Potter (1953) no $P_i$ was found. Kielly and Kielly (1951), Potter, Simonson and Siekevitz (1953) and others have shown that the " ATPase " activity of freshly-prepared mouse and rat liver mitochondria suspended in isotonic sucrose is extremely low or completely absent (latent " ATPase "). The " ATPase " of the fresh mouse liver mitochondria under the present experimental conditions were not completely latent as shown in Fig. 1 (solid lines) but agreed well with the findings of others (Wade and Jones, Jr., 1956).

FIG. 1.—The effect of γ-globulin on mouse liver mitochondrial ATPases and their activation by 2 : 4 DNP.

Isolation of mitochondria in 0·25 M sucrose, incubation at 27° C. in Tris buffer (0·05 M) containing KCl (0·1 M), $MgSO_4$ (0·005 M) and ATP (0·005 M). pH 7·4. 0·05 mg. mitochondrial N per flask.

●———● controls, ○———○ 2 : 4-DNP ($10^{-4}$ M) present, ●- - - -● γ-globulin (2 per cent) present, ○- - - -○ γ-globulin + 2 : 4-DNP present.

Under the conditions of the earlier experiments (Emmelot, Bos and Brombacher, 1956) we were unable to find any activation of the tumour mitochondrial " ATPases " in the presence of 2 : 4-DNP and only a two-fold activation of the corresponding activities of the liver mitochondria was observed. The 2 : 4-DNP activation has now been studied with small amounts of sucrose mitochondria and using γ-globulin to afford some protection against the degenerative changes which are suffered by the mitochondria *in vitro*. In experiments with the sucrose mitochondria from 25 mg. of mouse liver, 2 per cent γ-globulin was found to suppress the spontaneous activation of the latent " ATPases " as shown in Fig. 1 (compare Wade and Jones, Jr., 1956). It has further been observed that 2 : 4-DNP ($10^{-4}$ M) activates the " ATPases " of our liver preparations to a greater extent in the presence of γ-globulin than in its absence (Fig. 1).

The active ATPases of the sucrose-EDTA mitochondria from the adrenal cortex carcinoma T17572 could be also suppressed by γ-globulin but only during the first 10 minutes of incubation (Fig. 2). 2 : 4-DNP ($10^{-4}$ M) did activate the tumour mitochondrial ATPases under the latter conditions and caused a phosphate

FIG. 2.—The effect of γ-globulin on tumour mitochondrial ATPases (mouse adrenal cortex carcinoma T 17572) and their activation by 2 : 4-DNP.

Isolation in 0·25 M sucrose containing 0·001 M EDTA (pH 7·4) 0·05 mg. mitochondrial N per flask. Compare Fig. 1.

release which was of the same order as that shown by the mitochondria in the absence of the protective protein. The less active ATPases of the sucrose-EDTA mitochondria from the hepatoma T28012 were markedly lower in the presence of γ-globulin during the whole 20-minute incubation period. 2 : 4-DNP activated the ATPases to a marked extent under the latter conditions (Fig. 3).

An unexpected stimulating effect on the mitochondrial ATPases was found when the suspension medium was supplemented with 2 per cent aged human serum albumin. This preparation had been kept in our laboratory for some five years at room temperature. The aged albumin was found to activate the liver mitochondrial ATPases in the same manner as 2 : 4-DNP. The activation of the tumour mitochondrial ATPases was especially significant after the first 5 minutes and decreased in proportion when the incubation was carried out longer (Fig. 4). 2 : 4-DNP did not activate the ATPases of the liver or tumour mitochondria further when the aged protein was present. The effect of the preparation is contrary to the protection that may be expected to arise from the addition of fresh albumin and that was indeed also found. When incubated separately with ATP the aged albumin did not cause $P_i$ release and neither did it affect the pH of the suspension medium during the incubation. The latter was rigorously controlled since the ATPase activities of the tumour mitochondria, like those of liver mitochondria (Swanson, 1956), have been found (unpublished observations) to vary with the pH of the medium. After cold extraction with petrol-ether the aged albumin still exhibited its effect. The extract was evaporated but the small amount of fatty residue did not produce any activation of the ATPases of the liver mitochondria.

FIG. 3.—The effect of γ-globulin on tumour mitochondrial ATPases (mouse hepatoma T 28012) and their activation by 2 : 4-DNP.

Isolation in 0·25 M sucrose containing 0·001 M EDTA (pH 7·4) 0·04 mg. mitochondrial N per flask. Compare Fig. 1.

FIG. 4.—The effect of aged albumin on tumour mitochondrial ATPases (mouse adrenal cortex carcinoma T 17572) and their activation by 2 : 4-DNP.

Isolation in 0·25M sucrose containing 0·001 M EDTA (pH 7·4) 0·12 mg.N per flask.

●———● controls, ○———○ 2 : 4-DNP ($10^{-4}$ M) present, ●- - - -● aged albumin present (2 per cent), ○- - - -○ aged albumin plus 2 : 4-DNP present.

*Phosphate release from ADP and ATP catalysed by mitochondrial sub-fractions*

Myokinase can be obtained in a soluble form by disrupting liver mitochondria (Kielley and Kielley, 1951, 1953), while the "ATPase" remains bound to the sedimentable mitochondrial débris. Any appreciable $P_i$ release from ADP will thus only be possible when the soluble and sedimented parts of the disrupted liver mitochondria are incubated together. This is clearly illustrated by the experiments listed in Table VII which were carried out with mouse liver mitochondrial sub-fractions obtained by disrupting the mitochondria, as described under Material and Methods, and centrifugation for 30 minutes at 20,000 $\times$ g at pH 7·4. Centrifugation at pH 5·0 did not change the results. The synergistic action between the two sub-fractions in liberating $P_i$ from ATP may be readily explained on account of the myokinase activity of the soluble fraction which removes the ADP that is inhibitory to the "ATPase" (Kielley and Kielley, 1953).

*Table VII.—Phosphate release from ADP and ATP Catalyzed by Liver Mitochondrial Sub-fractions.*

1 = sedimented fraction and 2 = soluble fraction of disrupted mitochondria ; 1 + 2 = fractions combined after separation. In Experiments *a* and *b* centrifugation was carried out at pH 7·4, in Experiment *c* at pH 5·0 ; compare under Materials and Methods. Experiments *b* and *c* were performed with the same batch but not the same amount of disrupted mitochondria.

| | | µg. $P_i$ released per flask after 20 minutes (by fraction) | | | mg. N/flask | |
|---|---|---|---|---|---|---|
| Experiment | Substrate | 1 | 2 | 1+2 | 1 | 2 |
| *a* | ADP | 31 | 6 | 104 | 0·27 | 0·10 |
| | ATP | 102 | 16 | 165 | | |
| *b* | ADP | 10 | 8 | 125 | 0·16 | 0·16 |
| | ATP | 155 | 32 | 232 | | |
| *c* | ADP | 10 | 4 | 98 | 0·26 | 0·16 |
| | ATP | 127 | 10 | 200 | | |

Similar experiments were also carried out with the sub-fractions prepared from the tumour mitochondria. The first three experiments listed in Table VIII show that no synergistic action in liberating $P_i$ either from ADP or ATP could be observed on mixing the two sub-fractions. This was apparently due to the lack of separation since the myokinase came down with the sedimented fraction. This occurred irrespective of the fact whether the centrifugation was carried out at pH 5·0 or 7·4. After centrifugation at 7·4 the supernatant was somewhat turbid. The pH was therefore changed to 5·0 ; this resulted in a clear supernatant.

Judging from the nitrogen content of two sub-fractions it appeared also that only a small part of the particulate material became soluble in the case of the tumour mitochondria.

Evidently, either the tumour mitochondria contained less soluble material or the disruption was far from complete. The latter may not be unreasonable since the size of the tumour mitochondria was usually smaller than that of the liver mitochondria. Therefore hepatoma mitochondria were used which were approximately equal in size to the liver mitochondria. In this case (T28012)

TABLE VIII.—*Phosphate Release from ADP and ATP Catalyzed by Tumour Mitochondrial Sub-fractions*

(Compare Table VII)

Experiment *a* : Mitochondria isolated in sucrose-EDTA. Separation of sub-fractions by centrifugation at pH 7·4 ; Experiment *b* : *ibidem* at pH 5·0 ; Experiments *c* and *d* : mitochondria isolated in the absence of EDTA, separation of sub-fractions at pH 5·0 ; Experiments *e* and *f* : same batch of mitochondria, isolated in sucrose-EDTA, separation of sub-fractions at pH 5·0 ; during the treatment with distilled water $10^{-4}$ M Na-dodecyl-sulphate was present in Experiment *f*.

| Experiment | Mitochondria from | Substrate | μg. $P_i$ released per flask after 20 minutes (by fraction) | | | mg. N/flask | |
|---|---|---|---|---|---|---|---|
| | | | 1 | 2 | 1+2 | 1 | 2 |
| *a* | T5358 (testicular carcinoma) | ADP | 71 | 22 | 92 | 0·15 | 0·05 |
| | | ATP | 140 | 26 | 157 | | |
| *b* | T17572 (adrenal cortex carcinoma) | ADP | 132 | 0 | 141 | 0·23 | —* |
| | | ATP | 176 | 0 | 180 | | |
| *c* | R733 (ovarian tumour rat) | ADP | 125 | 2 | 143 | 0·14 | 0·03 |
| | | ATP | 200 | 3 | 212 | | |
| *d* | T28012 (hepatoma) | ADP | 52 | 5 | 85 | 0·21 | 0·04 |
| | | ATP | 112 | 5 | 119 | | |
| *e* | ,, ,, | ADP | 35 | 9 | 62 | 0·12 | 0·02 |
| | | ATP | 88 | 20 | 116 | | |
| | ,, ,, | ADP | 32 | 3 | 72 | 0·16 | 0·08 |
| | | ATP | 120 | 32 | 152 | | |

* Sample lost.

not withstanding the small amount of soluble nitrogen some synergism was found between the two sub-fractions in liberating $P_i$ from ADP but not such an effect was seen with ATP. The results of the latter experiment could be readily reproduced with other tumours of the same strain but the mitochondria from another hepatoma (T26473) yielded negative results.

The final elucidation of this problem awaits further investigation of tumour mitochondria disrupted by other means and the application of higher centrifugal forces.

Our type of liver mitochondrial sub-fractions was apparently different from that of Keilley and Kielley (1953). These authors found that on disrupting liver mitochondria in a Waring blendor, about 60–70 per cent of the ATPase activity released was recovered in the supernatant after centrifugation at 20,000 × g and that 80–90 per cent of this activity was in turn recovered in the pellet after centrifugation at 110,000 × g. Since our ATPase preparation from the disrupted liver mitochondria was almost completely sedimentable at 20,000 × g, it is evident that in the present work the mitochondria were not broken down to such small fragments as in the experiments of the American authors.

*Oxidative phosphorylation by tumour mitochondria*

It has not been possible to obtain satisfactory oxidation of BHB by the tumour mitochondria which show high ATP- and DPN-splitting activities (sarcoma

UV256, adrenal cortex carcinoma T17572, etc.) The only possible exception is provided by the mitochondria from the hepatoma T26473 which may be classified however on other grounds as the least representative of this group of tumours (Emmelot and Bos, 1955*a*, 1956*b*).

Most of the other tumour mitochondria exhibiting smaller ATP- and DPN-splitting activities than the former, show a satisfactory oxygen uptake in the presence of D-BHB but are unable to oxidize L-BHB. This oxidation of D-BHB, like that of citric acid cycle intermediates as shown for other tumour mitochondria (Williams-Ashman and Kennedy, 1952 ; Kielley, 1952 ; Lindberg *et al.*, 1953 ; Potter and Siekeviz, 1951) is accompanied by phosphorylation. In the absence of fluoride low P : O ratios, but in the presence of fluoride (0·01 M) P : O ratios of over 2 were obtained as illustrated in Table IX. The action of fluoride in inhibiting the tumour mitochondrial myokinase and " ATPase ", which enzymes would otherwise make any net phosphate uptake difficult to demonstrate, is very clearly illustrated in the experiment with the mitochondria from T26473 (compare Table V). Some control experiments on the effect of fluoride on the phosphate release from ATP and ADP by tumour mitochondria over a 15-minute period did indeed show that the "ATPases" were inhibited for 50-60 per cent and the myokinases for 80–100 per cent.

In a few cases the mitochondria from the sarcoma UV256 effected a small oxidation of pyruvate in the presence of L-malate and DPN. The P : O ratio found was half of that obtained with the mitochondria from the ovarian tumour T5441 or the testicular tumour T26554. This again demonstrated the difference in biochemical integrity between the representative of the two groups of tumour mitochondria.

TABLE IX.—*Phosphorylation Accompanying the Oxidation of* DL-*BHB and Pyruvate by Tumour Mitochondria*

Experimental conditions as in Table II, except that tumour mitochondria (approximating 0·5–0·7 mg. N) 21 μ-moles $P_i$ and 16 μ-moles NaF were present. In four experiments 2 μ-moles pyruvate and 0·5 μ-mole L-malate were added.

| Mitochondria from | Substrate | $-\Delta P_i$ μ-moles | $-\Delta 0$ μ-atoms | P : O |
|---|---|---|---|---|
| T 5358 (testicular carcinoma) | DL-BHB | 4·3 | 1·9 | 2·3 |
| T26473 (hepatoma) | ,, | 6·9 | 2·4 | 2·9 |
| T28102 (hepatoma) | ,, | 7·7 | 3·7 | 2·1 |
| T 5441 (ovarian tumour) | Pyruvate + L-malate | 4·0 | 2·5 | 1·6 |
| UV 256 (sarcoma) | ,, ,, | 1·2 | 1·5 | 0·8 |
| T26554 (testicular tumour) | ,, ,, | 10·7 | 5·8 | 1·8 |
| CBA71 (hepatoma) | ,, ,, | 10·8 | 5·4 | 2·0 |

### SUMMARY

It has been shown that sucrose-EDTA prepared mitochondria from certain tumour strains uncouple the phosphorylations normally associated with the oxidation of D-β-hydroxybutyrate by liver mitochondria, when both types of mitochondria are incubated together in the presence of DL-β-hydroxybutyrate and DPN. Consequently, the ATP-dependent oxidation of L-β-hydroxybutyrate, and in analogy that of octanoate, is abolished in a combined liver-tumour mitochondrial system. The tumour mitochondria possessed very pronounced " ATP-

ase " and myokinase activities as shown by the release of inorganic phosphate from ADP. In contrast, mitochondria from a number of other tumours, which have been shown earlier to be biochemically more intact than the former mitochondria (e.g. " ATPases ", DPNases and TPNases less active and no inhibition of the ATP- or DPN-dependent oxidative processes of the liver mitochondria) released much less inorganic phosphate from ADP. The latter tumour mitochondria yielded P : O ratios from 2–3 in the presence of DL-$\beta$-hydroxybutyrate or pyruvate when fluoride was added.

Myokinase and " ATPase " activities could easily be separated by disruption of liver mitochondria and centrifugation but in the case of tumour mitochondria results were unsatisfactory.

Addition of 2 per cent fresh human serum $\gamma$-globulin suppressed the " ATPase " activities of both liver and tumour mitochondria. Under these conditions an activation of the tumour mitochondrial " ATPases " by 2 : 4-dinitrophenol could readily be demonstrated. An aged preparation of human serum albumin, unlike fresh albumin, activated the enzymes of both types of mitochondria very significantly.

### REFERENCES

AISENBERG, A. C. AND POTTER, V. R.—(1956) *J. biol. Chem.*, **220,** 831.

CHAPPELL, C. D. AND PERRY, S. V.—(1954) *Nature*, **173**, 1094.

EMMELOT, P. AND BOS, C. J.—(1955*a*) *Rec. Trav. chim. Pays-Bas*, **74**, 1343.—(1955*b*) *Biochim. Biophys. Acta*, **18**, 281.—(1956*a*) *Ibid.*, **19**, 565.—(1956*b*) *Enzymologia*. In press.

*Idem*, BOS, C. J. AND BROMBACHER, P. J.—(1956) *Brit. J. Cancer*, **10**, 188.

*Idem* AND BROMBACHER, P. J.—(1956*a*) *Biochim. Biophys. Acta*, **21**, 581.—(1956*b*) *Ibid.*, **22**, 487.

FISKE, C. H. AND SUBBAROW, Y.—(1929) *J.biol. Chem.*, **81**, 629.

KIELLEY, R. K.—(1952) *Cancer Res.*, **12**, 124.

KIELLEY, W. W. AND KIELLEY, R. K.—(1951) *J. biol. Chem.*, **191**, 485.—(1953) *Ibid.*, **200**, 213.

LEHNINGER, A. L. AND GREVILLE, G. D.—(1953) *Biochim. Biophys. Acta*, **12**, 188.

LINDBERG, O., LJUNGGEN, M., ERNSTER, L. and RÉVÉSZ, L.—(1953) *Exp. Cell Res.*, **4**, 243.

POTTER, V. R. AND SIEKEVITZ, P.—(1951) ' Phosphorus Metabolism '. Vol. II, p. 665. Baltimore (Johns Hopkins Press).

*Idem*, SIMONSON, H. C. AND SIEKEVITZ, P.—(1953) *J. biol. Chem.*, **205**, 893.

SIEKEVITZ, P. AND POTTER, V. R.—(1953) *Ibid.*, **200**, 187.

SWANSON, M. A.—(1956) *Biochim. Biophys. Acta*, **20**, 85.

THIN, C. AND ROBERTSON, A.—(1952) *Biochem. J.*, **51**, 218.

WADE, R. AND JONES, Jr., H. W.—(1956) *J. biol. Chem.*, **220**, 547.

WILLIAMS-ASHMAN, H. G. AND KENNEDY, E. P.—(1952) *Cancer Res.*, **12**, 416.